# A gene expression signature distinguishes innate response and resistance to proteasome inhibitors in multiple myeloma

**DOI:** 10.1038/bcj.2017.56

**Published:** 2017-06-30

**Authors:** A K Mitra, T Harding, U K Mukherjee, J S Jang, Y Li, R HongZheng, J Jen, P Sonneveld, S Kumar, W M Kuehl, V Rajkumar, B Van Ness

**Affiliations:** 1 Department of Genetics, Cell Biology and Development, University of Minnesota, Minneapolis, MN, USA; 2 School of Statistics, University of Minnesota, Minneapolis, MN, USA; 3 Genome Analysis Core, Center for Individualized Medicine, Mayo Clinic, Rochester, MN, USA; 4 Division of Bioinformatics and Biostatistics, Department of Health Science Research, Mayo Clinic, Rochester, MN, USA; 5 Biomarker Discovery Program, Center for Individualized Medicine, Mayo Clinic, Rochester, MN, USA; 6 Department of Hematology, Erasmus MC Cancer Institute, Rotterdam, The Netherlands; 7 Division of Hematology, Department of Internal Medicine, Mayo Clinic, Rochester, MN, USA; 8 Genetics Branch, Center for Cancer Research, National Cancer Institute, Bethesda, MD, USA

## Abstract

Extensive interindividual variation in response to chemotherapy is a major stumbling block in achieving desirable efficacy in the treatment of cancers, including multiple myeloma (MM). In this study, our goal was to develop a gene expression signature that predicts response specific to proteasome inhibitor (PI) treatment in MM. Using a well-characterized panel of human myeloma cell lines (HMCLs) representing the biological and genetic heterogeneity of MM, we created an *in vitro* chemosensitivity profile in response to treatment with the four PIs bortezomib, carfilzomib, ixazomib and oprozomib as single agents. Gene expression profiling was performed using next-generation high-throughput RNA-sequencing. Applying machine learning-based computational approaches including the supervised ensemble learning methods Random forest and Random survival forest, we identified a 42-gene expression signature that could not only distinguish good and poor PI response in the HMCL panel, but could also be successfully applied to four different clinical data sets on MM patients undergoing PI-based chemotherapy to distinguish between extraordinary (good and poor) outcomes. Our results demonstrate the use of *in vitro* modeling and machine learning-based approaches to establish predictive biomarkers of response and resistance to drugs that may serve to better direct myeloma patient treatment options.

## Introduction

Wide interindividual variation in response to chemotherapy is a major limitation in achieving consistent therapeutic effect in many cancers, including multiple myeloma (MM), the second-most common hematologic malignancy with an estimated 30 330 new cases (~2% of all new cancer cases) and 12 650 estimated deaths in 2016 (NCI-SEER (The Surveillance, Epidemiology, and End Results program of the National Cancer Institute) Cancer statistics).^1, 2, 3, 4^ Such heterogeneity in response to treatment is governed in large part by the underlying molecular characteristics of the tumor, including differences in the expression of genes involved in mechanisms of chemoresistance.^5, 6, 7^ Deciphering key changes in gene expression levels underlying personalized sensitivity to chemotherapy is therefore essential to predict the efficacy of anticancer drugs and to prevent delay in the selection of more effective alternative strategies

Proteasome inhibitors (PIs) are effective chemotherapeutic agents in the treatment of MM, used alone or in combination with other anticancer agents like alkylating agents, corticosteroids, immunomodulatory agents and histone deacetylase inhibitors.^7, 8, 9^ Bortezomib (Bz/Btz/Velcade) was the first PI to be approved by the US Food and Drug Administration for clinical application in 2003 for the treatment of relapsed and refractory MM.^10, 11, 12^ Other examples include second-generation PIs Carfilzomib (Cz/Cfz/Kyprolis), Oprozomib (Opz) and Ixazomib (Ix/MLN9708/Ninlaro).^7, 10, 13^ However, MM still remains mostly an incurable disease with 5-year survival rate of 48.5% (NCI-SEER (The Surveillance, Epidemiology, and End Results program of the National Cancer Institute) Cancer statistics). Furthermore, most MM patients ultimately undergo relapse, including patients with good response to initial treatment who eventually develop resistance to the therapy.^7^ Moreover, there are reports that patients who fail to respond to Bz may still respond to other PIs.^8, 14^

Most patients receive PIs in combination with other therapeutic agents; thus, the variability in PI response is difficult to assess. In addition, survival end points in clinical applications are measured in months to years, and hence developing prediction algorithms of response can be a long process.

Therefore, we utilized a collection of more than 50 human myeloma cell lines (HMCLs) generated through the immortalization of primary MM cells that represent a broad spectrum of the biological and genetic heterogeneity of MM^15^ to create an *in vitro* chemosensitivity profile in response to treatment with the four PIs: Bz, Cz, Ix and Opz as single agents. Then, we used machine learning-based computational approaches to identify gene signatures that could distinguish sensitive and resistant responses in cell lines. When applied to gene expression profiling (GEP) data of MM patients from four different PI-based clinical trials, our GEP model of response/resistance to PIs successfully distinguished differences in disease progression and distinguished extraordinary (good and poor) responses. Thus, these results can provide a PI treatment-specific predictor of clinically relevant outcomes that could affect therapeutic choices.

## Materials and methods

### Drugs

Bz (Takeda Pharmaceuticals Inc., Deerfield, IL, USA) was dissolved in serum-free RPMI-1640 (Lonza, Allendale, NJ, USA) and stored at −20 °C. Ix (Takeda), Cz and Opz (Amgen, Thousand Oaks, CA, USA) were dissolved in dimethyl sulfoxide (DMSO; Sigma-Aldrich, St Louis, MO, USA) and stored at −20 °C.

### Cell lines

Fifty HMCLs were procured from various institutions, established and characterized, and maintained in HMCL media with interleukin-6.^16^ Supplementary Table S1 provides the cytogenetic characteristics of the HMCLs.

### *In vitro* chemosensitivity assays

Cell cytotoxicity assays were performed on the HMCLs to create a drug sensitivity profile in response to treatment with increasing concentrations of Bz, Cz, Ix and Opz as single agents and half-maximal inhibitory concentration (IC_50_) values and area under the survival curve (AUSC) were calculated as described earlier.^16^ Briefly, cells were counted using Countess automated cell counter (Invitrogen, Carlsbad, CA, USA) and seeded in 96-well plates at a concentration of 4 × 10^5^ cells per ml. After 24 h, cells were treated with increasing concentrations of Bz, Cz, Ix and Opz as single agents. Cell viability assays were performed 48 h post treatment using CellTiter-Glo luminescent cell viability assay (Promega, Madison, WI, USA) according to the manufacturer’s instructions using Synergy 2 Microplate Reader (BioTek, Winooski, VT, USA) to generate survival curves. Percent survival values were normalized to untreated controls and IC_50_ values were determined by calculating the nonlinear regression using sigmoidal dose–response equation (variable slope). AUSC was calculated using trapezoidal rule and log_2_-transformed for further statistical analysis.^16^ Caspase-3/7 activity was evaluated using Caspase-Glo 3/7 Assay kit (Promega) on Synergy 2 Microplate Reader.

### Gene expression profiling

RNA was isolated from six most Ix-sensitive and six most Ix-resistant cell lines and RNA sequencing was performed on llumina HiSeq 2000 (Illumina, San Diego, CA, USA) using 50 bp paired-end protocol with depth of >20 million reads per sample. RNA-sequencing (RNA-seq) data from CD138-selected plasma cells were generated from MM patients enrolled in an ongoing phase-2 Ixazomib clinical trial at Mayo Clinic (Mayo-Ix; NCT01415882) that enrolled patients with relapsed myeloma who had less than six cycles of prior treatment with a Bz-based regimen and were not refractory to Bz^17^ (see Supplementary Methods for details).

Gene expression, treatment arm and outcome data on newly diagnosed myeloma patients enrolled in HOVON65/GMMG-HD4 trial (ISRCTN64455289; *n*=290) were downloaded from Gene expression omnibus (GEO) (GSE19784).^18^ The APEX data set (GSE9782; *n*=264) consists of bortezomib-based phase-2 and phase-3 relapsed and/or refractory myeloma clinical trials (The APEX phase-3 trial (039), a companion study (040), the SUMMIT (025) and CREST phase-2 trials (024)).^19^ For both these trials, Affymetrix, Santa Clara, CA, USA gene probe set analysis data (U133-Plus2.0 for HOVON65/GMMG-HD4 and HG-U133A/B for APEX) were available. Pretreatment RNA-seq data, treatment arm and clinical outcome information on CoMMpass (Relating Clinical Outcomes in MM to Personal Assessment of Genetic Profile) trial patients were downloaded from the MMRF (Multiple Myeloma Research Foundation) researcher gateway portal (IA7c release; https://research.themmrf.org). The CoMMpass Trial (NCT0145429), sponsored by MMRF, is a non-registrational, longitudinal study of 1000 newly diagnosed MM patients followed over the course of their disease, up to 8 years.^20^

### Bioinformatics and statistical analysis

All statistical analyses were performed using R software environment (https://www.r-project.org/) version 3.3.1 for statistical computing and graphics, and GraphPad Prism v7.0 (GraphPad Software, Inc., La Jolla, CA, USA). Spearman’s rank-order and Pearson’s product-moment correlation analyses were performed to compare the PI responses. All tests were two sided and *P*<0.05 was considered statistically significant. Gene expression data were preprocessed, log_2_-transformed and analyzed using Galaxy and Partek Genomics Suite v6.6 (Partek Inc., St Louis, MO, USA) to perform differential expression testing to identify gene expression signatures PI response (details in Supplementary Methods). Analysis of variance model or two-sided paired sample *t*-test was used to evaluate whether each gene is differentially expressed. Heatmaps were generated using unsupervised hierarchical clustering analysis based on the differentially expressed genes.

Supervised machine learning approaches construct algorithms that learn from training data, build models based on properties of training inputs and thus make learned predictions/decisions on new/test samples.^16, 21^ Random forest, a supervised ensemble machine learning algorithm, was used to establish the top differentially expressed genes as predictive GEP signatures of PI response.^21^ GEP data on top Ix-sensitive vs top Ix-resistant HMCLs (*n*=12) were used as ‘training data set’ to build random forest classification models (decision trees) and predict PI resistance of HMCLs (*n*=44) in the ‘test data set’, the mRNA-seq data obtained from the Keatslab repository (http://www.keatslab.org/data-repository). The average bootstrap prediction error was generated using repeated bootstrapping of the train data set with a *k*-fold cross-validation (*k*=100). The cross-validation error rate was used to evaluate the accuracy of the method.^16, 21^

The predictive GEP signature was then applied to the four different PI-based MM clinical trials and random survival forest estimation method for right censored data (randomForestSRC), another supervised machine learning decision-tree based algorithm, was used to predict probability of progression/event (0=censored; 1=progression) within the first 3 years for each myeloma patient.^22^ The predicted probability values derived using machine learning approaches (random forest and randomForestSRC) were rank-ordered and the predictions for the top (Q3) and bottom quantiles (Q1) were compared with observed PI response using Somers’ *D*_*xy*_ rank correlation^23^ (see details in Supplementary Methods).

Unsupervised *K*-means clustering was performed using the algorithm of Hartigan and Wong^24^ to identify clinically important *K*-subgroups based on our PI response GEP signature such that *N*/*K*~30 (*N*=total number of subjects in data set). Kaplan–Meier curves for survival were generated for the extraordinary PI response (good vs poor) clusters by computing progression-free survival (PFS) over time.^25^ The Kaplan–Meier survival curves were compared statistically using log-rank test and Cox proportion hazard test.^26^ Clusters with *n*<10 were combined for PFS comparisons.

Odds ratios (ORs) between observed clinical responses (available for APEX and CoMMpass data sets) vs extraordinary PI-response *K*-means clusters were computed using binomial logistic regression analysis (logit model).^27^

### Ingenuity pathway analysis

The differentially expressed genes were analyzed using Ingenuity Pathway Analysis (IPA) to identify canonical pathways, downstream effects, upstream regulators and causal networks and to perform predictive toxicology analysis using toxicogenomics approaches (IPA-Tox).^28^

## Results

### Wide variability in response to PI treatment

**Figure 1:**
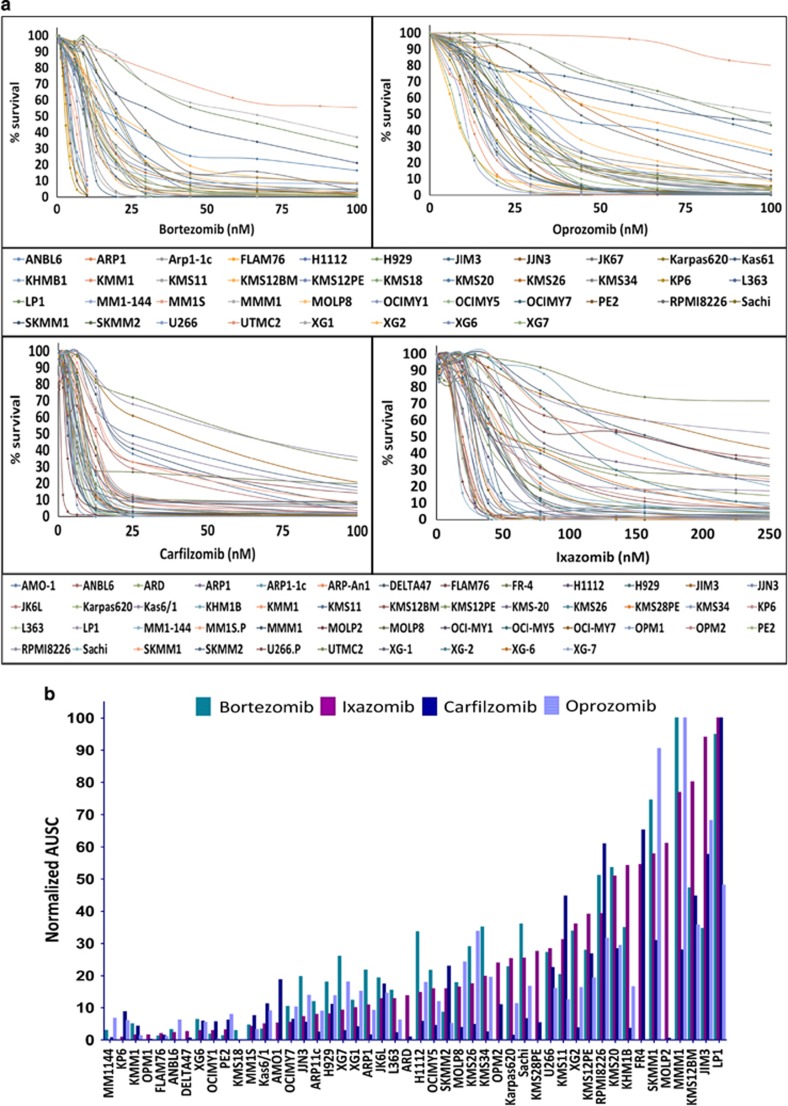

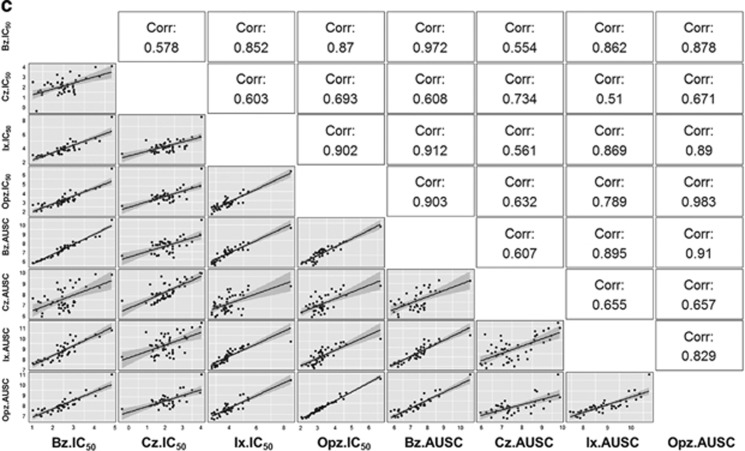
*In vitro* chemosensitivity profiles of human myeloma cell lines following proteasome inhibitor treatment. (**a**) Plots show survival compared with untreated control versus increasing concentration of bortezomib, oprozomib, ixazomib and carfilzomib. In (**b**), the AUSC was normalized and expressed as percentage of the largest value for each drug, shown for all cell lines treated with the four proteasome inhibitors. In (**c**), the scatterplot matrix is shown as a pairwise correlation of the natural log (Ln) of IC_50_ and AUSC values for the response to each PI drug. Scatterplot matrix was generated using the R graphing package ggplot2.

To model and characterize the interindividual variation in therapeutic response, we assessed the *in vitro* PI sensitivity of a large panel of HMCLs. Results of our cytotoxicity assays in 50 HMCLs showed a wide range of response to treatment with the four PIs (Bz, Cz, Ix and Opz) identifying some lines that are highly sensitive and some lines relatively refractory to PIs, as represented by the IC_50_ and AUSC plots (Figures 1a and b). In some cases, the AUSC provides a comparative measure of response through the higher test doses, especially if an IC_50_ value was not achieved. The summary table (Table 1) depicts the extensive variation in response to each of the PIs among the panel of HMCLs. Our caspase-3/7 activity data corroborated with cytotoxicity data (data not shown). Figure 1c shows a correlation matrix representing comparative analysis of IC_50_ values of four PIs across the HMCLs. Statistically significant (adjusted *P*-values of <0.001; Holm’s method) positive correlation was observed between IC_50_ values across the four PIs. Thus, in general, sensitivity was highly correlated among all four PIs, especially among the cell lines representing extraordinarily/exceptionally high and low responses. Therefore, we evaluated bortezomib and ixazomib as model proteasome inhibitor drugs for the gene expression signature-based prediction of extraordinary response in PI-inhibitor-based therapies.

However, we did find some examples of HMCLs that were highly sensitive to one PI, and comparably less sensitive to other PIs. This demonstrates tumor heterogeneity even in response to inhibitors of the same class, and further demonstrates some tumors refractory to one PI may still respond to another.

### Deriving a GEP-based signature profile of PI response

**Figure 2:**
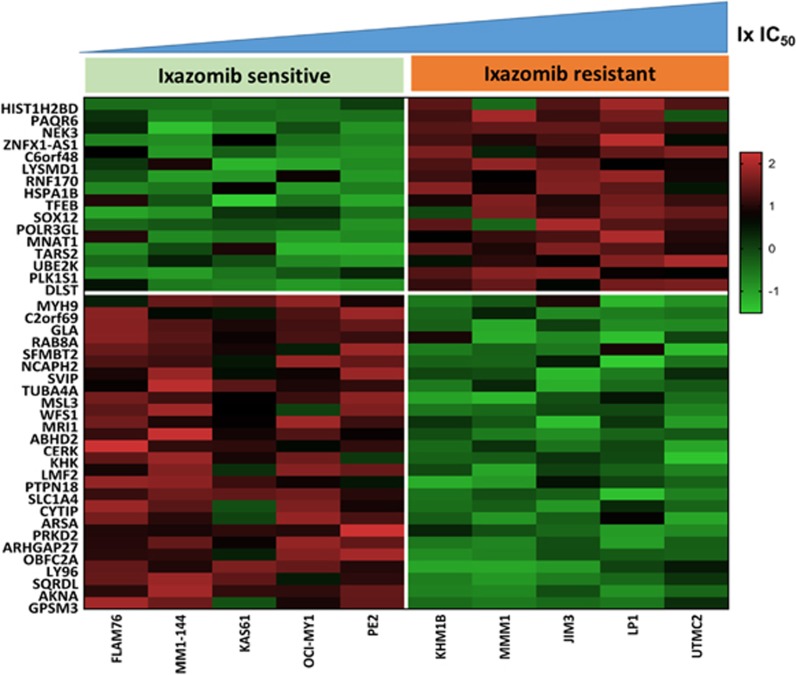
Heatmap representing differential gene expression between PI-sensitive and PI-resistant myeloma cell lines. Gene expression was *z*-score normalized (standardized: shifted to mean of 0 and scaled to s.d. of 1) and compared among the five most Ix-responsive and five least Ix-responsive cell lines. Heatmap was generated using the top 42 differentially expressed genes (|fold difference| >2; *P*<0.01). Columns are ordered by Ix IC_50_ of cell; genes are ordered by fold difference. Color indicates fold change between Ix-resistant and Ix-sensitive cell lines.

We performed differential gene expression analysis between 5 (top 10%) most Ix-sensitive and 5 (bottom 10%) most Ix-resistant cell lines. Notably, these cell lines showed the same relative sensitivity and resistance to all four proteasome inhibitors. RNA-seq data were prefiltered, normalized and used for further analysis (see Supplementary Methods). Differential gene expression analysis was performed using the remaining 7682 genes to identify GEP signatures that distinguish the highly sensitive from the highly resistant HMCLs. Results showed 506 genes differed significantly between the sensitive and the resistant groups (*P*<0.05; fold difference ≠1). In all, 141 genes showed |fold difference| >2 and *P*<0.05, whereas 42 genes, listed in Table 2, had *P*<0.01 (|fold difference| >2) (Figure 2). Subsequent analyses used the more stringent highly variable/differentially expressed 42-gene list. The purpose of this initial selection was to generate a list of genes that were highly variable/differentially expressed between PI-sensitive and PI-resistant cell lines and could then be used in further steps to derive the prediction model and for validation.

### GEP signature of PI response is predictive of *in vitro* PI chemosensitivity in HMCLs and progression in MM clinical trials

Our approach was to develop a novel methodology to predict extraordinary/exceptional (very high and very low) PI responses in patients using a myeloma cell line-based model system. The training data set comprised gene expression profiles of HMCLs that represent extremities of PI responses, whereas the initial validation/test data set was derived from an independent gene expression study on HMCLs (*n*=44) performed in the Keats laboratory at TGen laboratories (http://www.keatslab.org/data-repository). The final validation data set of human myeloma patients comprised four independent clinical trials (HOVON65/GMMG-HD4 (*n*=290); APEX (*n*=264); CoMMpass (*n*=765); and the Mayo-Ix trial (*n*=22)).

First, the random forest algorithm for classification was ‘trained’ using mRNA-seq data on the 42-gene signature from 6 most PI-sensitive and 6 most PI-resistant cell lines. The out-of-bag estimate, which is a measure of mean prediction error of the training model calculated by subsampling the training data set, was computed as 0%. Concurrently, the classification error rate derived from confusion matrix was also 0, thus validating the robustness of the training model. Supplementary Figure S1 provides the gene importance plot representing the most important genes within the 42-gene expression signature influencing PI response based on variable importance (VIMP) measure derived from random forest analysis. The probability scores of PI resistance were then calculated for each of the HMCLs obtained from the Keatslab data repository (‘test’ data set). The predicted probabilities of PI resistance were then rank-ordered and Somers’ QUOTE rank correlation analysis was performed between the top quantile (Q3) and bottom quantile (Q1) resistance probability values and observed PI chemosensitivity as a binary outcome (sensitive=0 vs resistance=1). Results revealed high positive Somers QUOTE rank correlation for the PI drug cytotoxicity parameters (IC_50_ and AUSC) (Table 3), indicating that our 42-gene GEP signature validated quite well in an independent data set of HMCLs. As the classification model was generated using HMCLs with top-6+bottom-6 Ix IC_50_ values as training data set, the test data set for Ix IC_50_ prediction included RNA-seq data on the remaining 32 cells lines only from an independent data set obtained from the Keatslab repository. Among these, Somers’ *c* value for correlation between observed Ix IC_50_ and response probabilities of the top 6 predicted Ix-resistant cell lines was 0.667 whereas it was 1.0 for the top 6 predicted Ix-sensitive lines. Somers’ *c* for the combined set of 12 predicted sensitive+resistant HMCLs was 0.743. Thus, the independent cell line test set showed very good correlation with the signature-derived predictor.

Although the training set showed good prediction capabilities in an independent set of HMCLs, we were particularly interested to determine whether it is able to stratify patient outcomes in multiple clinical trials. Four different PI-treating trials were examined (treatment details provided in Materials and Methods). Microarray gene expression data from clinical trials was mean-centered and scaled before analysis. The standardized transcriptomic profiling data from APEX trial was used as a training data set to perform random survival forest analysis to predict the percent probability of a progression/event within 3 years in the HOVON-GMMG-HD4, CoMMpass and Mayo-Ix clinical trials (test data sets). Somers’ QUOTE rank correlation analysis between the top and bottom quantiles of predicted percentage values from random survival forest model on test data and the progression index of the test data sets showed consistent positive values for the HOVON-GMMG-HD4, CoMMpass and Mayo-Ix clinical trials revealing high prediction accuracy of the random survival forest-based prediction model^22, 23^ (Table 4). Somers’ *c* for the training data set (APEX) was=0.852.

**Figure 3:**
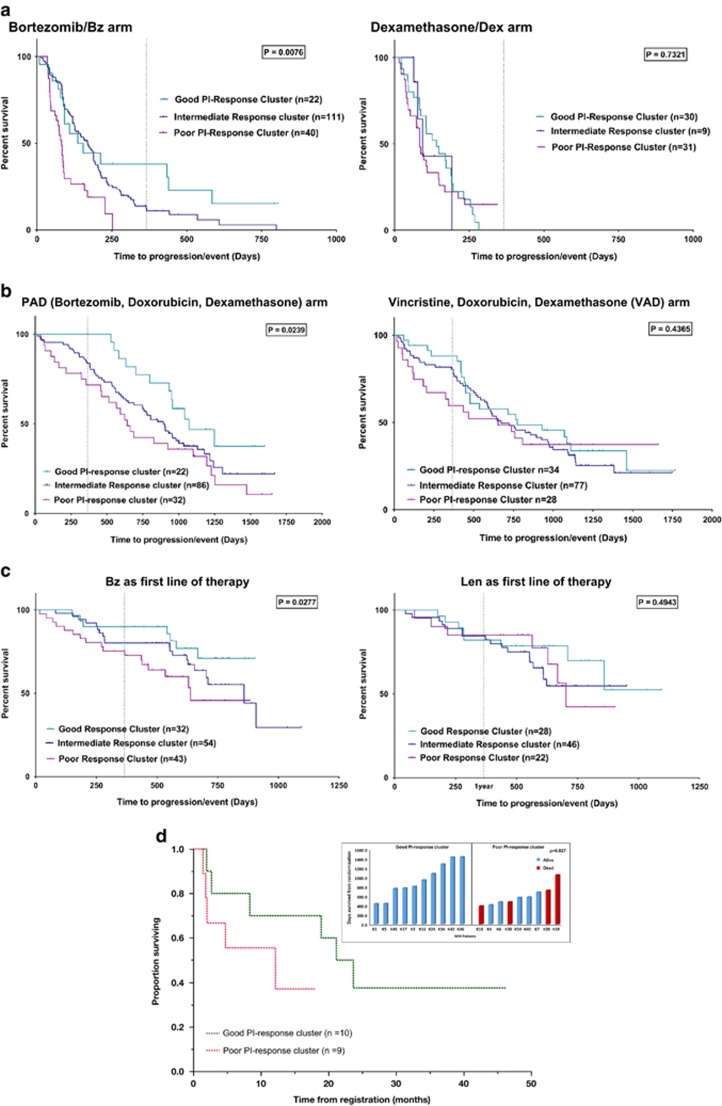
Plots showing stratification in progression-free survival (PFS) among MM patients on PI-based clinical trials in which the 42-gene model was used to assign extraordinary (good and poor) PI response. Kaplan–Meier survival curves in (**a**) APEX data set: bortezomib arm shows significant separation of PFS between clusters representing good vs poor outcomes, whereas the dexamethasone arm shows no stratification; (**b**) patients in the HOVON-GMMG-HD4 trial (Bz-treated/PAD and VAD arms) were assigned good versus poor PI response based on the 42 gene model. PFS curves for the interim analysis of the (**c**) CoMMpass trial (NCT0145429) patients administered Bz or Lenalidomide (Len) as first-line therapy and (**d**) the Mayo Clinic Ix-trial (NCT01415882). Patients were assigned good versus poor response based on the 42-gene model. Inset of (**d**) shows survival of each patient considered. Dashed line represents end of year 1 (365.25 days from randomization).

As we were particularly interested in our signature’s drug response performance, we chose to look at progression as one measure of response/nonresponse as well as associations that distinguish clinical definition of response, complete response versus nonresponse.^29^ *K*-means clustering was performed on 188 MM patients from the Bz treatment arm of the APEX data set^19^ to partition the samples into clusters/subgroups based on the expression of the 42 genes comprising the GEP signature of PI response. Results show significant differences in PFS between the signature-derived poor PI-response and good PI-response groups (hazard ratio (HR)=2.346; *P*=0.0076; Figure 3a). Conversely, no difference in PFS is observed between the *K*-means clusters when the 42-gene model was applied to the expression data of 76 patients in the dexamethasone arm of APEX phase-3 trial that compared single-agent Bz with high-dose dexamethasone (HR=1.1; *P*=0.732; Figure 3a), showing our GEP signature is drug specific. Concurrently, statistically significant association is observed between the *K*-means clusters and clinical response in the Bz arm (OR_responder vs nonresponder_=5.813; 95% confidence interval=1.833–20.007; *P*_OR_=0.0036) but not in the Dex arm (OR_responder vs nonresponder_=2.139; 95% confidence interval=0.753–6.326; *P*_OR_=0.158) of the APEX data set.

When applied to the gene expression data from HOVON-GMMG-HD4 clinical trial (*n*=290) that implemented a Bz-based drug regimen,^18^ the 42-gene signature shows statistically significant differences in PFS among K-means clusters representing good vs poor PI response in the Bz-treated PAD (bortezomib, doxorubicin and dexamethasone) arm (HR=2.161; *P*=0.024), whereas no difference in PFS is observed in the VAD (vincristine, doxorubicin and dexamethasone) arm (HR=1.282; *P*=0.437; Figure 3b). What is particularly striking is that within the first 1.5 years the predicted good response group in the PI-containing therapy had no progression events, whereas 35% of the predicted poor response group had progression events.

Clinical data were available on 765 patients from the CoMMpass study. A total of 253 patients used Bz as first-line therapy, alone or in combinations. Of these, 128 had RNA-seq and PFS data available. *K*-means clustering based on the expression of the 42 genes distinguishes between good and poor PFS subgroups of myeloma patients (HR=2.556; *P*=0.0277; Figure 3c). In addition, the *K*-means clusters are also found associated with clinical response (OR=5.20; 95% confidence interval=1.22 36.077; *P*_OR_=0.0453). In contrast, among the 96 patients using Lenalidomide (an immuno-modulatory drug) as first-line therapy, no association was observed between the good and poor *K*-means clusters and PFS (*P*=0.49; Figure 3c). Our results thus further validate the PI response specificity of our method.

Finally, we applied our algorithm based on the our 42-gene PI response signature to an ongoing clinical trial at Mayo Clinic that uses Ix-containing drug regimen. Interestingly, even with small numbers of patients available, our GEP-based PI response classifier distinguishes between the top (medium PFS=22.42 months) and bottom responders (medium PFS=12 155 months) in this Ix trial (risk ratio=2.5) (Figure 3d). Notably, all 10 patients in the good performance group were alive at the latest point of the interim analysis, whereas 4 out of 9 patients in the poor PI response cluster died (inset of Figure 3d).

### Ingenuity pathway analysis

Genes in the 42-gene GEP model were then analyzed using IPA to assign them to different functional networks. Out of the 42 genes, 12 overlapped with the top IPA network obtained from the analysis of direct and indirect relationships (score=26), as represented in Supplementary Figure S2a. Genes/molecules in this network include the 26s proteasome complex. Furthermore, when we used the IPA upstream regulator analysis, TFEB and CCND1 were identified as the top hits/factors that may control the genes and pathways highlighted by network analysis (Supplementary Figure S2b). The functional significance is discussed further in the Discussion.

## Discussion

Resistance to chemotherapy in cancers is a major limitation in achieving complete and sustained therapeutic effect, and may in fact lead to unwanted exposure to ineffective antitumor agents, thereby increasing the risk of negative side effects.^1^ MM is a mostly incurable disease with a median overall survival of 4.6 years despite significant improvement in treatment strategies over the past 12 years.^2, 3, 4, 30, 31^ Differential GEP studies have provided GEP signatures as a useful prognostic indicator of low- versus high-risk MM; however, none of these gene signatures were treatment specific.^15, 18, 32^ Furthermore, no study thus far has made use of the vast array of HMCLs as model system to generate drug chemosensitivity profile as a representation of the response variation in patient subtypes that may be used to derive a PI-specific GEP signature predictive of resistance and treatment outcomes.

In the current study, using a panel of 50 HMCLs, we successfully developed an *in vitro* chemosensitivity profile to four PI treatments. We focused on common features that distinguish the most and least responsive HMCLs, noting that these responses are very similar across all four PIs. However, several outliers (HMCLs that were highly resistant to one PI and highly sensitive to another) were noted. Interestingly, several cell lines with intermediate response to Bz or Ix were found highly responsive to Cz treatment. However, in general the ranked correlation with response was very similar among all four PIs, particularly those lines that were collectively the most responsive and least responsive to all four PIs (extraordinary responders). Further characterization of the outliers may reveal important features that better direct which PI is most effective.

Using transcriptomic profiling of PI-sensitive vs PI-resistant HMCLs followed by extensive machine learning-based response prediction, we demonstrate here a gene expression signature that distinguishes strong sensitivity versus strong resistance to the four PIs when used in MM. Importantly, this signature was not applied across the full range of responses, but was very effective in identifying extraordinary (good versus poor) response. We also observed that this 42-gene signature of innate PI response is different from a signature of *acquired* PI response that we generated by comparing GEP between PI-sensitive and subsequent resistant outgrowths of Bz-resistant MM cell lines (unpublished data).

Despite the small numbers of cell lines used to generate the 42-gene signature, when applied to clinical trials on MM patients undergoing PI-based therapies, our GEP signature was remarkably correlated with extraordinary PI response/resistance, could predict early or late progression events and efficiently clustered patient responses into favorable (response or complete response) vs unfavorable (nonresponse) outcome subgroups. Notably, the GEP signature did not stratify response to non-PIs in the APEX, HOVON and CoMMpass trials, suggesting PI treatment specificity, not simply high- versus low-risk tumor biology irrespective of drug treatment regimen as has been previously reported.^32^

Several genes included in our GEP signature have been previously implicated with prognosis and disease progression in myeloma. For example, the cell cycle regulator CCND1 (cyclin-D1) has been reported as a strong prognostic predictor in multiple studies.^15, 16^ HSPA1B is an NRF2-mediated oxidative stress response gene that was also found significant (*P*<0.001; |fold change| >2) in our earlier study that compared differential responsiveness with Bz *in vitro*.^33^ Abnormal levels of serum bone Gla protein have been shown to play important role in the inhibition of bone formation in the occurrence of bone lesions in MM that may have prognostic implications.^34^ Wolfram syndrome 1 encodes an endoplasmic reticulum (ER) transmembrane protein that plays a role in regulation of ER stress signaling and in the pathogenesis of diseases involving chronic, unresolvable ER stress.^35^ Furthermore, Wolfram syndrome 1 is a downstream transcriptional target of the gene X-box binding protein (XBP1), a bZIP transcription factor that regulates unfolded protein response and ER stress-induced apoptosis.^35, 36^ Higher expression of XBP1 pathway genes is associated with better outcome/response in PI-treated MM patients, whereas loss of XBP1 has been shown to induce PI resistance.^36, 37^ Interestingly, we found that the unspliced transcript of XBP1 (Xbp1u) had ~1.5 times lower expression in Ix-resistant cell lines when compared with Ix-sensitive cell lines. The transcription factor EB (TFEB) is a master gene for lysosomal biogenesis that drives expression of a large subset of autophagy genes leading to activation of autophagic cell death of myeloma cells owing to metabolic cell stress.^38^ RNF170 encodes an ER membrane ubiquitin ligase that mediates ubiquitination and degradation and plays a key role in cell signaling.^39^ Given previous reports on the association of these genes to myeloma biology and outcomes, we considered their collective inclusion in our unsupervised clustering further validation of their role in PI response. Although individually associated with MM biology, our GEP signature suggests a combination of effectors influence response to PIs. Additional studies will be required to functionally validate the combined effects of these genes towards response to PI-based therapy.

Earlier, we demonstrated that gene expression signatures may be used to identify secondary therapies in PI-resistant MM using *in silico* predictions that were confirmed *in vitro*.^33, 40^ In 2014, the NCI initiated the Exceptional Responders Initiative to understand the molecular basis of exceptional response to chemotherapy in cancer patients enrolled in clinical trials.^41^ The primary goal of the study is to identify molecular features in malignant tissue that may aid to predict response to same or similar drugs. On similar lines, our work should serve as resource to use machine learning-based approaches for the personalized prediction of exceptional chemoresistance and to eventually identify signatures of drug combination regimens that may effectively reverse drug resistance by predicting drugs for various subpopulations/subclones of tumors based on pharmacogenomic signature profiles.

## Figures and Tables

**Table 1:** Numerical summaries of chemosensitivity parameters in HMCLs

*Proteasome inhibitor*	*Mean (nm)*	*Minimum (nm)*	*Median (nm)*	*Maximum (nm)*
Bortezomib_ IC_50_	17.1	2.8	11.7	124.3
Carfilzomib_ IC_50_	10.9	0.7	7.1	55.3
Ixazomib_ IC_50_	155.3	15.1	42.1	4757.9
Oprozomib_ IC_50_	45.8	7.6	23.7	776.0
Bortezomib_ AUSC	2700.1	319.6	1524.0	38 974.0
Carfilzomib_ AUSC	3503.4	375.5	1448.5	19 097.0
Ixazomib_ AUSC	10 030.0	1702.0	6456.0	46 494.0
Oprozomib_ AUSC	5017.0	1050.0	2885.0	59 917.0

Abbreviations: AUSC, area under the survival curve; HMCL, human myeloma cell line; IC_50_, half-maximal inhibitory concentration.

**Table 2:** List of genes most significantly associated with proteasome inhibitor (PI) resistance (|fold difference| >2; *P*<0.01)

*No.*	*Gene ID*	P*-value*	*Fold difference (sensitive vs resistant)*	*Fold difference (description)*
1	*SLC1A4*	0.00004	2.695	Sensitive up vs resistant
2	*NEK3*	0.00006	−2.544	Sensitive down vs resistant
3	*GLA*	0.00007	2.073	Sensitive up vs resistant
4	*AKNA*	0.00020	4.043	Sensitive up vs resistant
5	*ARHGAP27*	0.00035	3.599	Sensitive up vs resistant
6	*LY96*	0.00045	3.796	Sensitive up vs resistant
7	*DLST*	0.00070	−2.001	Sensitive down vs resistant
8	*MSL3*	0.00132	2.237	Sensitive up vs resistant
9	*SQRDL*	0.00134	3.906	Sensitive up vs resistant
10	*NCAPH2*	0.00206	2.129	Sensitive up vs resistant
11	*PLK1S1*	0.00262	−2.035	Sensitive down vs resistant
12	*MRI1*	0.00284	2.448	Sensitive up vs resistant
13	*TARS2*	0.00294	−2.083	Sensitive down vs resistant
14	*OBFC2A*	0.00307	3.756	Sensitive up vs resistant
15	*RAB8A*	0.00319	2.097	Sensitive up vs resistant
16	*ABHD2*	0.00363	2.475	Sensitive up vs resistant
17	*LMF2*	0.00364	2.558	Sensitive up vs resistant
18	*C6orf48*	0.00367	−2.462	Sensitive down vs resistant
19	*TUBA4A*	0.00400	2.189	Sensitive up vs resistant
20	*HSPA1B*	0.00467	−2.335	Sensitive down vs resistant
21	*TFEB*	0.00471	−2.302	Sensitive down vs resistant
22	*RNF170*	0.00504	−2.34	Sensitive down vs resistant
23	*SOX12*	0.00569	−2.281	Sensitive down vs resistant
24	*ZNFX1-AS1*	0.00604	−2.482	Sensitive down vs resistant
25	*C2orf69*	0.00622	2.038	Sensitive up vs resistant
26	*PTPN18*	0.00634	2.635	Sensitive up vs resistant
27	*PRKD2*	0.00641	3.5	Sensitive up vs resistant
28	*KHK*	0.00662	2.484	Sensitive up vs resistant
29	*PAQR6*	0.00710	−3.645	Sensitive down vs resistant
30	*HIST1H2BD*	0.00763	−4.514	Sensitive down vs resistant
31	*CERK*	0.00776	2.481	Sensitive up vs resistant
32	*UBE2K*	0.00806	−2.048	Sensitive down vs resistant
33	*LYSMD1*	0.00814	−2.379	Sensitive down vs resistant
34	*GPSM3*	0.00832	5.309	Sensitive up vs resistant
35	*MNAT1*	0.00906	−2.144	Sensitive down vs resistant
36	*WFS1*	0.00911	2.397	Sensitive up vs resistant
37	*MYH9*	0.00923	2.011	Sensitive up vs resistant
38	*CYTIP*	0.00925	2.855	Sensitive up vs resistant
39	*SVIP*	0.00928	2.148	Sensitive up vs resistant
40	*ARSA*	0.00931	3.3	Sensitive up vs resistant
41	*SFMBT2*	0.00947	2.111	Sensitive up vs resistant
42	*POLR3GL*	0.00958	−2.251	Sensitive down vs resistant

Differential gene expression analysis was performed to compare gene expression profiles of 5 (top 10%) most ixazomib (Ix)-sensitive and 5 (bottom 10%) most Ix-resistant cell lines.

These 42 genes were used as gene expression profiling (GEP) signature of PI resistance to stratify PI response in test data sets (*in vitro* and among patients).

**Table 3:** Summary of correlation between predicted probabilities of PI resistance and observed PI cytotoxicity values

	*Somers'* c			*Spearman's ρ*	
	*c_Q3_*	*c_Q1_*	*C_Q3+Q1_*	*Spearman_Q3+Q1_*	P
Bz_IC_50_	0.643	0.786	0.852	0.748	0.00036
Cz_IC_50_	0.714	0.524	0.750	0.563	0.00981
Opz_IC_50_	0.667	0.944	0.802	0.626	0.00548
Bz_AUSC	0.643	0.786	0.852	0.736	0.00050
Cz_AUSC	0.595	0.667	0.712	0.601	0.00507
Ix_AUSC	0.857	0.889	0.927	0.765	0.00009
Opz_AUSC	0.667	0.786	0.813	0.630	0.00509

Abbreviations: AUSC, area under the survival curve; Bz, bortezomib; Cz, carfilzomib; IC_50_, half-maximal inhibitory concentration; Ix, ixazomib; Opz, oprozomib; PI, proteasome inhibitor.

Random forest classification model was generated using human myeloma cell lines (HMCLs) with top-6+bottom-6 Ix IC_50_ values as training data set.

Predicted probability values of HMCLs in the test data set were rank-ordered and Somers’ *D*_*xy*_ rank correlation analysis was performed between the top quantile (Q3) and bottom quantile (Q1) resistance probability values observed PI chemosensitivity as a binary outcome (sensitive=0 vs resistance=1).

Spearman’s rank-ordered correlation was performed in cell lines representing Q3 and Q1 probabilities of resistance and corresponding cytotoxicity values.

**Table 4:** Summary of Somers’ *D*_*xy*_ rank correlation analysis between predicted probability values of progression (derived from random survival forest model) and the progression index of MM patients from PI-based clinical trials (test data sets)

	*Somers'* c		
	*c*_*Q3*_	*c*_*Q1*_	*C*_*Q3+Q1*_
HOVON-GMMG-HD4 (PAD arm)	0.596	0.599	0.561
CoMMpass–Bz first-line therapy	0.705	0.469	0.595
CoMMpass–Len first-line therapy	0.320	0.203	0.262
Mayo-Ix	0.500	0.833	0.680
APEX-Dex arm	0.365	0.467	0.431

Abbreviations: Bz, bortezomib; Cz, carfilzomib; Dex, dexamethasone; Ix, ixazomib; Len, lenalidomide; MM, multiple myeloma; PAD, bortezomib, doxorubicin and dexamethasone; PI, proteasome inhibitor.

Transcriptomic profiling data from APEX trials were used as training data set.
