# Supplementary material for: A gene expression signature distinguishes innate response and resistance to proteasome inhibitors in multiple myeloma
Source: Blood Cancer J. 2017 Jun 30;7(6):e581–. doi: 10.1038/bcj.2017.56 (PMC5520403; doi:10.1038/bcj.2017.56)
Supplement: Supplementary Information [file bcj201756x1.docx]

**Supplementary Methods**

**RNA-sequencing**

**HMCLs**

High quality RNA was extracted from HMCLs using QIAshredder and RNeasy kit (Qiagen). RNA concentration and integrity were analyzed using the Nanodrop-8000 and Agilent 2100 Bioanalyzer and stored at -80°C. RNA integrity number/RIN>8 was considered suitable for RNA-seq analysis. RNA-seq library construction was performed using the Illumina TruSeq RNA sample Preparation kit v2. The libraries were size selected to generate inserts of ~200bp and RNA sequencing was performed using llumina’s HiSeq 2000 next-generation high-throughput sequencing system using 50bp paired-end protocol with depth of >20million reads-per-sample. Average quality scores were well above Q30 for all libraries in both R1 and R2. Data was normalized and FPKM values were used in further analysis using a combination of Galaxy data analysis software and Partek Genomics Suite. GEP vs *in vitro* chemo-sensitivity data was then used to identify gene expression signatures associated with PI response.

**Mayo Clinic Ixazomib trial**

Bone marrow samples were obtained from patients prior to enrolment. RNA extraction was performed on CD138-selected plasma cells using AllPrep DNA/RNA mini kit (Qiagen). 100ng high-quality RNA were used for RNA-seq library construction using the Illumina TruSeq RNA sample Preparation kit v2 (Illumina Inc., San Diego, CA, USA). The libraries were size selected to generate inserts of ~200bp and RNA sequencing was performed using llumina’s HiSeq 2000 next-generation high-throughput sequencing system using 109bp paired-end protocol with depth of ~100million reads per sample (Illumina Inc., San Diego, CA, USA).

**RNA-Seq data processing**

Gene expression data was pre-processed using Galaxy, an open source, web-based platform that provides tools necessary to create and execute RNA-seq analysis. Briefly, an RNA-seq data analysis pipeline was developed using Galaxy that performs quality control (QC) check on the RNA-seq raw reads using FastQC tool, trims the reads to remove base positions that have a low median (or bottom quartile) score **and then** uses **Tophat2**tool to map processed RNA-seq reads to the hg19 human genome build. Estimated insert sizes were derived using Picard's CollectInsertSizeMetrics tool on this initial tophat2 run which was used to calculate mean inner distance between mate pairs (Mean= estimated_insert-size - 2*read_length). Tophat2 was then re-run using correct mean value and finally **Cufflinks**tool was used on these datasets to assemble the reads into transcripts.

Prior to analysis, the processed transcripts were filtered using the following criteria: genes with variance=0, FPKM<1 and mean FPKM<5 were removed.

**Random Forest**

Random forests are ensemble machine learning methods for classification/regression that depend on creation of an ensemble (collection) of decision trees to predict the modal class in classification models or the mean prediction in regression models (1). The random forest algorithm has two distinct steps: 1. For a specific set of variables (genes in our scenario), creation of a classifier or regressor based on decision tree method; 2. creation of a forest (ensemble) of such trees through a two level randomization process involving selection of variables for any tree and then randomizing the split of variables through random re-sampling (bootstrap step) of the data(1).

In our analysis, we employed a random forest-based classification model approach that used PI-sensitive vs PI-resistance information of the training dataset (6 most Ix-sensitive vs 6 most Ix-resistant HMCLs) to predict PI- resistance in the test dataset of HMCLs (mRNA-seq data obtained from the Keatslab repository, http://www.keatslab.org/data-repository) using our 42 gene GEP signature of PI-response. Our analysis parameters were: number of trees/ntree = 500; Total number of variables = 42. The average bootstrap prediction error was generated using repeated bootstrapping of the train dataset. For any given train data-set, we used K-fold cross validation (k=100) for generating a cross validation error during the training process. To generate the distribution of the error rates, we re-sampled the train data-set randomly with replacement. Using these random subsets of train data-set, we generated a vector of cross validation errors by repeated training of the random forest model. We used 500 re-samples of the train set to generate 500 cross validation errors. The vector of CV error was used to generate a robust estimation of average CV error.

Furthermore, the out-of-bag (OOB) error or out-of-bag estimate rate was used to evaluate the accuracy of the method. Importantly, the Out*-*of-bag estimates help avoid the need for cross-validation or an independent validation dataset in random forests (1).

**Random Survival Forest**

Random survival forests (RSF) extend the random forest method to survival data which are usually censored and not completely observed (2). RSF is a random forest-based ensemble class prediction method for right censored survival data such as patient survival data, disease progression data, and drug treatment data (2). Censoring creates issues in standard regression analysis by introducing bias due to truncation of the underlying data distribution. This requires the use of proportional hazard based non-parametric (Kaplan-Meier) or semi-parametric (Cox proportional hazard) models. Nonlinearity of relationships need to be usually captured through nonlinear transformation of data in standard survival models. However, RSF takes care of these issues in a more robust manner for prediction purposes. In RSF, the first level of randomization of bootstrapped data sampling is inherited as is from random forest method. In the second step, survival trees are grown on the randomized bootstrapped samples by splitting nodes such that it maximizes the difference between the survival measure (response measure) of the two daughter nodes in the survival tree. Once the ensemble of trees is grown, an ensemble cumulative baseline survival hazard is computed by aggregating (averaging) survival estimates from the ensemble of survival trees (2).

Thus, RSF is performed based on a combination of the random forest method (ensemble of decision trees) and the Kaplan-Meier (KM) estimator for survival data. The tree based survival function is computed by using the Kaplan Meier (KM) estimator for a decision tree’s terminal node. To be precise, if $Y_{h}^{\left( n \right)}\left( t \right)$ be the number of individuals in terminal node $h$ of a random survival forest at time $t$, i.e., the number of individuals in a terminal node who are at risk, but have neither experienced an event nor have been censored, and if $N_{h}^{\left( n \right)}(t)$ be the number of events in the interval $(0,t]$ for all cases in $h$, then the KM estimator for cases within $h$ is given by the following equation.

$${\hat{\boldsymbol{S}}}_{\boldsymbol{h}}\left( \boldsymbol{t} \right)\boldsymbol{=}\prod_{\boldsymbol{s\leq t}} \left( \boldsymbol{1-}\frac{\boldsymbol{d}\boldsymbol{N}_{\boldsymbol{h}}^{\left( \boldsymbol{n} \right)}\left( \boldsymbol{s} \right)}{\boldsymbol{Y}_{\boldsymbol{h}}^{\left( \boldsymbol{n} \right)}\left( \boldsymbol{t} \right)} \right)$$

The survival function for individual $i$ is the average of $B$ bootstrapped KM estimator for the terminal node where individual $i$ belongs and is given by the equation (2).

$$\hat{\boldsymbol{S}}\left( \boldsymbol{t} | \boldsymbol{x}_{\boldsymbol{i}} \right)\boldsymbol{=}\frac{\boldsymbol{1}}{\boldsymbol{B}}\sum_{\boldsymbol{b=1}}^{\boldsymbol{B}} {\hat{\boldsymbol{S}}}_{\boldsymbol{b}}\left( \boldsymbol{t} | \boldsymbol{x}_{\boldsymbol{i}} \right)\boldsymbol{=}\frac{\boldsymbol{1}}{\boldsymbol{B}}\sum_{\boldsymbol{b=1}}^{\boldsymbol{B}} \sum_{\boldsymbol{h}} \boldsymbol{I(}\boldsymbol{x}_{\boldsymbol{i}}\boldsymbol{\in h)} \hat{\boldsymbol{S}_{\boldsymbol{h}}}\boldsymbol{(t)}$$

Hence, random survival forests retain the essence and advantages of random forest, but is customized to handle right censored survival data as in our case.

The models were estimated using the R package ***randomForestSRC*** (https://cran.r-project.org/web/packages/randomForestSRC/randomForestSRC.pdf). Our analysis parameters were: number of trees/ntree = 500; Total number of variables = 42; Analysis = RSF; Family = Surv; Splitting rule = logrank.

**Somers’** $\boldsymbol{D}_{\boldsymbol{xy}}$ **rank correlation**

The evaluation of random forest and random survival forest prediction methods was performed using Somers’ $D_{xy}$ rank correlation between predicted percentage values on test data and the binary outcome (chemosensitivity/progression index) of the test data.

Somers’ $D_{xy}$is a measure of correlation between a variable x and a binary (0-1) variable y and is given by the following equation (3):

$$\boldsymbol{D}_{\boldsymbol{xy}}\boldsymbol{=2\times}\left( \boldsymbol{c-0.5} \right)$$

Where, $c$ is equivalent to the area under the curve (AUC) of the receiver operating characteristics (ROC) corresponding to the predicted values and the binary outcome (0 or 1) of the test data. The Somers’ $D_{xy}$ indicates the incremental predictive accuracy beyond random assignment and can vary between$[-1,1]$. A high positive Somers’ $D_{xy}$ rank correlation value indicates high prediction accuracy of any predictive model.

The Somers’ $D_{xy}$ rank correlation of predicted values from the random survival forest model was computed using the *somers2* function from the package *Hmisc* in R (https://cran.r-project.org/web/packages/Hmisc/Hmisc.pdf).

**References**

1. Breiman L. Random Forests. *Mach Learning* 2001; **45**: 5-32.

2. Ishwaran H, Kogalur UB, Blackstone EH, Lauer MS. Random Survival Forests. *The Annals of Applied Statistics* 2008; **2**: 841-60.

3. Somers RH. A new asymmetric measure of association for ordinal variables. *Am Sociol Rev* 1962;: 799-811.
