# Supplementary material for: A gene expression signature distinguishes innate response and resistance to proteasome inhibitors in multiple myeloma
Source: Blood Cancer J. 2017 Jun 30;7(6):e581–. doi: 10.1038/bcj.2017.56 (PMC5520403; doi:10.1038/bcj.2017.56)
Supplement: Supplementary Table [file bcj201756x2.docx]

**Table S1**. List of Human Myeloma Cell Lines (HMCLs) included in this study.

[Translocations and cyclin D expression (TC)-based classification system- 4=4;14 translocation involving MMSET/FGFR3; 11/6/12=Cyclin D1/3/2 IgH translocation; M = MAF.IgH; M.L,M.K &M.0=rearrangement with IgL,IgK, or no Ig; D2 = CYCLIN D2 expression without primary IgH TLC; 0 = no Ig TLC; low expression of all 3 Cyclin D genes]

| **Cell lines** | **Number of Chromosomes** | **Translocations and cyclin D expression (TC)-based classification system** | **TC Group code** |
| --- | --- | --- | --- |
| H929 | 45 | 4;14 translocation involving MMSET/FGFR3 | 4 |
| JIM3 | 61 | 4;14 translocation involving MMSET/FGFR3 | 4 |
| Kas6/1 | 70 | 4;14 translocation involving MMSET/FGFR3 | 4 |
| KMS18 | 74 | 4;14 translocation involving MMSET/FGFR3 | 4 |
| KMS26 | 75 | 4;14 translocation involving MMSET/FGFR3 | 4 |
| KMS28PE | 43 | 4;14 translocation involving MMSET/FGFR3 | 4 |
| KMS34 | 71 | 4;14 translocation involving MMSET/FGFR3 | 4 |
| LP1 | 80 | 4;14 translocation involving MMSET/FGFR3 | 4 |
| OPM1 | 74 | 4;14 translocation involving MMSET/FGFR3 | 4 |
| OPM2 | 74 | 4;14 translocation involving MMSET/FGFR3 | 4 |
| PE2 | 72 | 4;14 translocation involving MMSET/FGFR3 | 4 |
| UTMC2 | 77 | 4;14 translocation involving MMSET/FGFR3 | 4 |
| XG7 | 43 | 4;14 translocation involving MMSET/FGFR3 | 4 |
| KMM1 | 80 | Cyclin D3 IgH translocation | 6 |
| FLAM76 | 42 | Cyclin D1 IgH translocation | 11 |
| H1112 | 46 | Cyclin D1 IgH translocation | 11 |
| Karpas620 | 68 | Cyclin D1 IgH translocation | 11 |
| KMS12BM | 77 | Cyclin D1 IgH translocation | 11 |
| KMS12PE | 77 | Cyclin D1 IgH translocation | 11 |
| MMM1 | 47 | Cyclin D1 IgH translocation | 11 |
| MOLP8 | 86 | Cyclin D1 IgH translocation | 11 |
| OCIMY7 | 78 | Cyclin D1 IgH translocation | 11 |
| SKMM2 | 37 | Cyclin D1 IgH translocation | 11 |
| U266P/VR | 39 | Cyclin D1 IgH translocation | 11 |
| XG1 | 44 | Cyclin D1 IgH translocation | 11 |
| AMO1 | 75 | Cyclin D2 IgH translocation | 12 |
| DELTA47 | 45 | CYCLIN D2 expression without primary IgH TLC | D2 |
| FR4 | 100 | CYCLIN D2 expression without primary IgH TLC | D2 |
| JK6L | 50 | CYCLIN D2 expression without primary IgH TLC | D2 |
| KHM1B | 59 | CYCLIN D2 expression without primary IgH TLC | D2 |
| KMS20 | 41 | CYCLIN D2 expression without primary IgH TLC | D2 |
| KP6 | 47 | CYCLIN D2 expression without primary IgH TLC | D2 |
| MOLP2 | NA | CYCLIN D2 expression without primary IgH TLC | D2 |
| OCIMY1 | 49 | CYCLIN D2 expression without primary IgH TLC | D2 |
| ANBL6 | 82 | MAF.IgH translocation | M |
| ARD | 48 | MAF.IgH translocation | M |
| ARP1 | 48 | MAF.IgH translocation | M |
| ARP11c | 48 | MAF.IgH translocation | M |
| JJN3 | 60 | MAF.IgH translocation | M |
| MM1S/VR | 44 | MAF.IgH translocation | M |
| OCIMY5 | 46 | MAF.IgH translocation | M |
| SACHI | 42 | MAF.IgH translocation | M |
| SKMM1 | 79 | MAF.IgH translocation | M |
| L363 | 46 | No Ig rearrangement | M_0 |
| KMS11 | 70 | MAF.IgH translocation; 4;14 translocation involving MMSET/FGFR3 | M_4 |
| RPMI8226 | 60 | rearrangement with IgL, | M_L |
| XG2 | 49 | rearrangement with IgL, | M_L |
| XG6 | 77 | rearrangement with IgL, | M_L |
