# Supplementary material for: A gene expression signature distinguishes innate response and resistance to proteasome inhibitors in multiple myeloma
Source: Blood Cancer J. 2017 Jun 30;7(6):e581–. doi: 10.1038/bcj.2017.56 (PMC5520403; doi:10.1038/bcj.2017.56)
Supplement: Supplementary Figure [file bcj201756x3.ppt]

## Slide 1
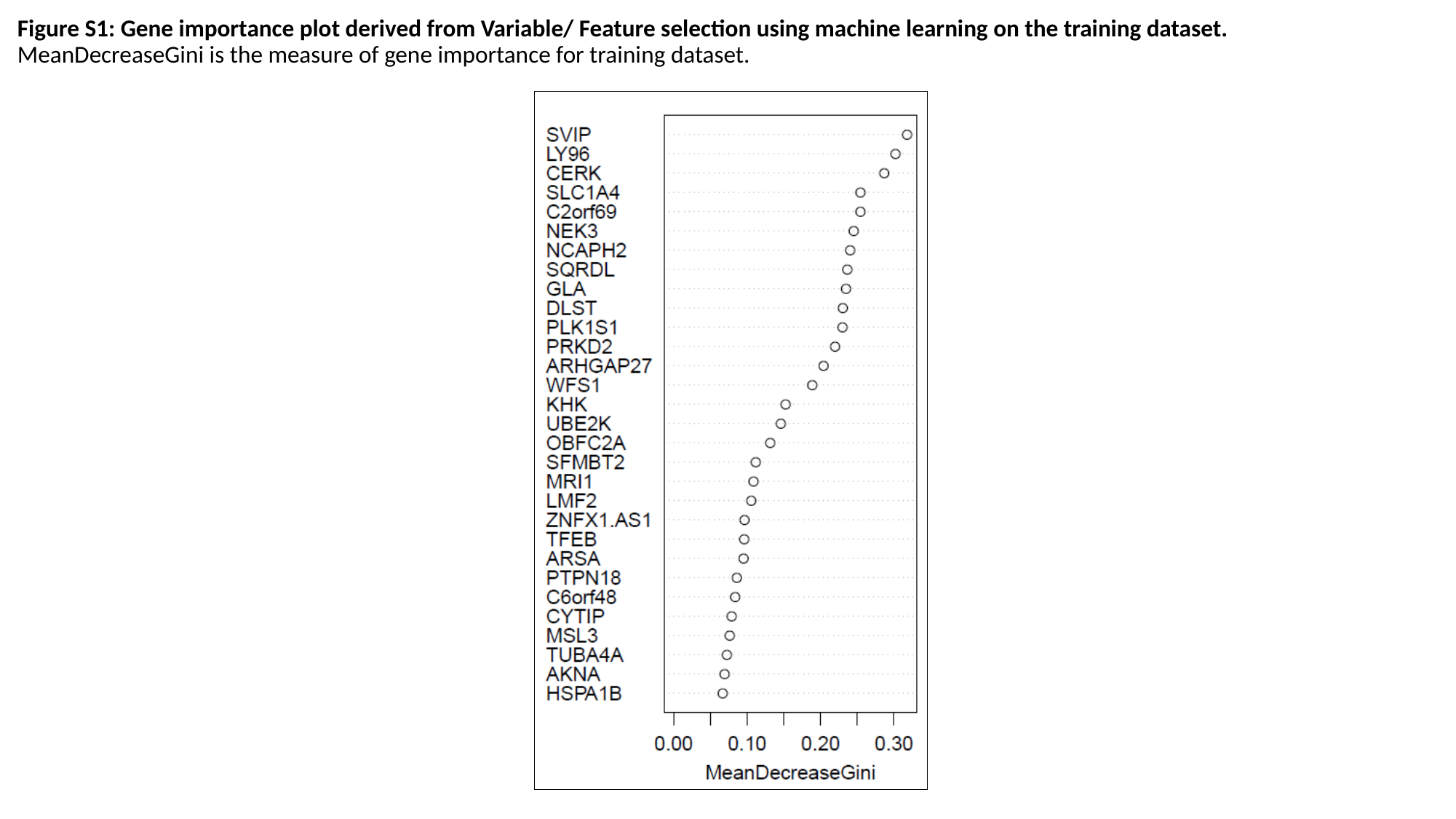

Figure S1: Gene importance plot derived from Variable/ Feature selection using machine learning on the training dataset.
MeanDecreaseGini is the measure of gene importance for training dataset.

## Slide 2
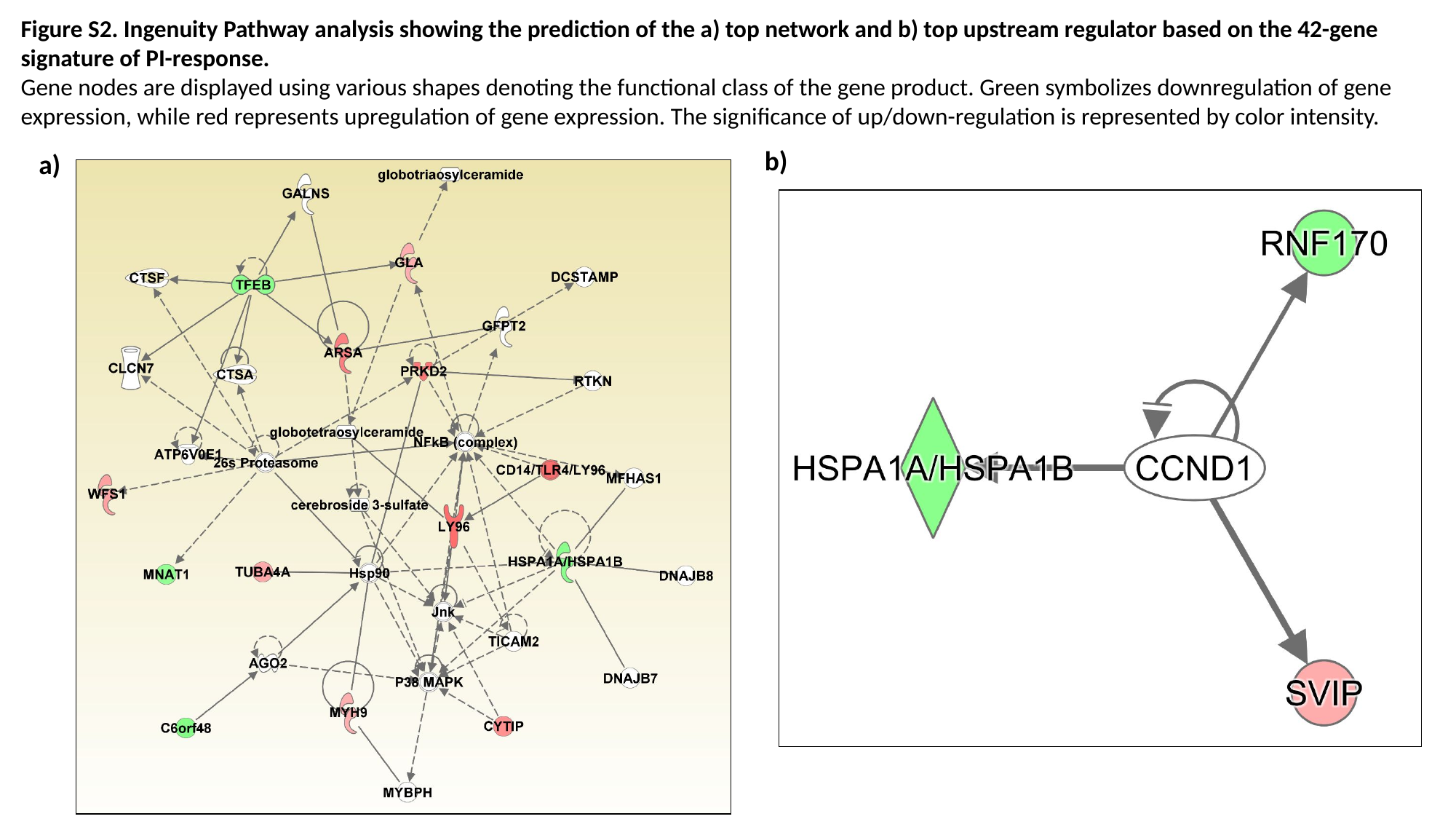

Figure S2. Ingenuity Pathway analysis showing the prediction of the a) top network and b) top upstream regulator based on the 42-gene signature of PI-response.
Gene nodes are displayed using various shapes denoting the functional class of the gene product. Green symbolizes downregulation of gene expression, while red represents upregulation of gene expression. The significance of up/down-regulation is represented by color intensity.
b)
a)
